# Exploration of Optimal pH in Hypothermic Machine Perfusion for Rat Liver Grafts Retrieved after Circulatory Death

**DOI:** 10.3390/jcm12113845

**Published:** 2023-06-04

**Authors:** Sodai Sakamoto, Hiroki Bochimoto, Kengo Shibata, Nur Khatijah Mohd Zin, Moto Fukai, Kosei Nakamura, Takahisa Ishikawa, Masato Fujiyoshi, Tsuyoshi Shimamura, Akinobu Taketomi

**Affiliations:** 1 Department of Gastroenterological Surgery 1, Hokkaido University Graduate School of Medicine, Sapporo 060-0815, Japan; soudai114@gmail.com (S.S.);; 2 Department of Cell Physiology, The Jikei University School of Medicine, Tokyo 105-8461, Japan; botimoto@jikei.ac.jp (H.B.);; 3 Gastroenterological Surgery 1, Hokkaido University Hospital, Sapporo 060-8648, Japan; 4 Division of Organ Transplantation, Hokkaido University Hospital, Sapporo 060-8648, Japan

**Keywords:** liver, transplantation, machine perfusion, hypothermic machine perfusion, donation after circulatory death, DCD, mitochondria, pH, osmium-maceration

## Abstract

Ex vivo hypothermic machine perfusion (HMP) is a strategy for controlling ischemia-reperfusion injury in donation after circulatory death (DCD) liver transplantation. The pH of blood increases with a decrease in temperature and water dissociation, leading to a decrease in [H^+^]. This study aimed to verify the optimal pH of HMP for DCD livers. Rat livers were retrieved 30 min post-cardiac arrest and subjected to 3-h cold storage (CS) in UW solution (CS group) or HMP with UW-gluconate solution (machine perfusion [MP] group) of pH 7.4 (original), 7.6, 7.8, and 8.0 (MP-pH 7.6, 7.8, 8.0 groups, respectively) at 7–10 °C. The livers were subjected to normothermic perfusion to simulate reperfusion after HMP. All HMP groups showed greater graft protection compared to the CS group due to the lower levels of liver enzymes in the former. The MP-pH 7.8 group showed significant protection, evidenced by bile production, diminished tissue injury, and reduced flavin mononucleotide leakage, and further analysis by scanning electron microscopy revealed a well-preserved structure of the mitochondrial cristae. Therefore, the optimum pH of 7.8 enhanced the protective effect of HMP by preserving the structure and function of the mitochondria, leading to reduced reperfusion injury in the DCD liver.

## 1. Introduction

Expanding donor sources in liver transplantation is a global challenge [1]. As a solution to this donor shortage, liver transplantation using expanded criteria donor (ECD) grafts, such as donation after cardiac death (DCD) and donors with fatty liver disease, has been explored [2,3,4,5]. The frequency of complications, including delayed graft function, primary nonfunction, and bile duct complications, is high in liver transplantation using DCD liver grafts, and more than half of them cannot be used without modification [6]. The efficiency of ex vivo machine perfusion (MP) for DCD liver grafts has been studied using hypothermic machine perfusion (HMP) [7] and normothermic MP [8]. However, a standardized protocol for MP in liver transplantation has not yet been established.

The UW-gluconate solution (Belzer-MPS^®^), which is used in liver [9,10] and kidney [11] HMP, consists of phosphate buffers. Given that the pH of a phosphate buffer is barely influenced by the temperature, the pH of the UW-gluconate solution also varies only minimally with temperature [12]. In contrast, the histidine-imidazole buffer derived from hemoglobin, which is one of the strongest buffering systems, exhibits pH changes with temperature (Rosenthal factor: ΔpH/ΔT = −0.0147) [13]. The pH of pure water is not always 7.0 because the dissociation of water molecules changes with temperature. Accordingly, the pH is calculated from the proton concentration and the ionic product of water. It is important to understand that the pH alone is not a measure of electrochemical neutrality without considering temperature, because the neutrality of the acid-base equilibrium is dependent on the balance between the concentration of protons and hydroxyl ions [12]. Even after excluding gas exchange, the pH of blood also changes similarly to pure water due to the effect of imidazole.

Poikilotherms, such as frogs, adjust their physiological pH according to temperature through respiration, which keeps the pCO_2_ constant [14]. This adjustment process is known as the alpha-stat strategy. On the other hand, the process of maintaining a constant pH, also through respiration, regardless of temperature, is known as the pH-stat strategy. The concept of alpha-stat and pH-stat has been investigated, particularly in anesthesia management during cardiovascular surgery and hypothermic management after cardiac arrest [15]. The pH adjustment in the hypothermic management for cerebral ischemia protection, such as for major vascular surgery, is performed by controlling the pCO_2_. It has been shown that pH-stat, in which pCO_2_ is increased at low temperatures and adjusted to approximately pH 7.4, reduces vascular resistance due to increased CO_2_ concentration [16]. However, acidosis is known to cause tissue damage by mitochondrial failure due to free radicals injuring the components of the respiratory chain [17]. Regardless, pH control, an essential factor in organ HMP, still needs to be explored. Although there are some reports on renal MP [18], limited research has been conducted on liver preservation [19].

The most effective approach for HMP in the liver, including pH-stat and alpha-stat, is yet to be determined in cases where a phosphate buffer-based perfusion solution is used. Investigating this issue will aid in improving and standardizing perfusion solutions for MP in the liver. We hypothesized that the optimal acid-base equilibrium for cells is determined not by the absolute concentration of protons but by the ratio of protons and hydroxyl ions. The present study aimed to determine the optimal pH of HMP for DCD liver grafts and to elucidate the mechanism of graft protection. We investigated the optimal pH for HMP in liver transplantation using isolated perfused rat liver apparatus (IPRL) simulating normothermic reperfusion.

## 2. Materials and Methods

### 2.1. Chemicals and Reagents

Unless otherwise noted, all chemicals and reagents were purchased from Wako Pure Chemical Co., Ltd. (Osaka, Japan). The UW solution (Belzer UW^®^) and UW-gluconate solution (Belzer MPS^®^) were purchased from Bridge to Life Ltd. (Northbrook, IL, USA).

### 2.2. Animals

Male Wistar rats (8 weeks old) were purchased from Sankyo Labo Service Corporation, Inc. (Tokyo, Japan) and housed for at least 1 week, with three or fewer rats per cage under constant climatic conditions with free access to standard laboratory feed (MF; Oriental Yeast, Tokyo, Japan) and water. The composition of the feed was as follows: total lipids 5.6 g, tocopherol 9.1 mg, ascorbate 5.5 mg, and phospholipids 816 mg per 100 g. This study was performed in accordance with the Institutional Guide of Hokkaido University for the Care and Use of Laboratory Animals with the approval of the Institutional Review Board (No. 17-0032).

### 2.3. Procedure

Rats (9–12 weeks, 220–300 g) were anesthetized using isoflurane inhalation without fasting. Cardiopulmonary arrest (CPA) was initiated via diaphragmatic transection. After 30 min of CPA administration, the portal vein was cannulated using a 16-G catheter (NIPRO, Osaka, Japan). The liver was flushed via the portal vein with 50 mL cold saline (4 °C) containing 20,000 IU/L of sodium heparin (Mochida Pharmaceutical Co., LTD, Tokyo, Japan). The graft was then flushed and replaced with 20 mL of ice-chilled Belzer UW^®^ or UW-MPS^®^ solution. The bile duct was cannulated with polyethylene tubing PE-10 (BD Intramedic, Clay Adams, NJ, USA), and the liver was removed.

### 2.4. Study Protocol

In the control (CT) group, the liver without CPA was reperfused immediately after procurement on an IPRL (*n* = 6). In the other groups, all animals were subjected to 30 min of CPA, and the livers were removed thereafter. In the CS group, livers were stored in UW solution at 4 °C for 3 h (*n* = 6). The HMP group was further divided into four groups. Livers were treated with 3 h of HMP with UW-MPS^®^ without pH adjustment in the MP group (*n* = 6), while in the MP-pH groups, livers were subjected to 3 h of HMP with UW-MPS^®^ with pH adjustment to 7.6, 7.8, and 8.0 at 7–10 °C (MP-pH 7.6, MP-pH 7.8, and MP-pH 8.0 groups, respectively) (each *n* = 6). The pH during HMP was monitored using a pH meter (LAQUAact, HORIBA, Kyoto, Japan) throughout the HMP period in all groups. The values of pO_2_ and pCO_2_ were measured using a RAPIDLab 348EX (SIEMENS, Munich, Germany).

### 2.5. Conditions of the HMP

The livers were perfused with 300 mL of UW-MPS^®^ at 7–10 °C and a constant pressure of 4–6 cmH_2_O in a recirculating system. The perfusates of MP-pH 7.6, 7.8, and 8.0 were adjusted to pH 7.6, 7.8, and 8.0 ± 0.05, respectively, by the addition of NaOH (2 mol/L) before the commencement of perfusion and thereafter, if required. The oxygen concentration of the perfusate was controlled by the oxygen pressure exposed to the silicon tubing in the perfusion circuit. Optimal oxygen tension was maintained throughout the HMP (450 < pO_2_ < 550 mmHg). We measured portal vein pressure (PVP) and portal flow and calculated portal vein resistance (PVR) during 180 min of HMP.

### 2.6. IPRL Settings for the Simulating Reperfusion

IPRL conditions were set according to the method described by Bessems et al. [20] with slight modifications to the method [21]. Briefly, livers were perfused with 300 mL of Krebs–Henseleit bicarbonate buffer (KHB) supplemented with glucose (10 mM) and sodium taurocholate (0.3 mM) at 37 °C in a recirculating system. PVP was maintained at 12 cmH_2_O in all groups except the CT group, which was maintained at 8 cmH_2_O. The pH of KHB was adjusted to 7.35–7.45 at 37 °C by bubbling CO_2_ before reperfusion. The oxygen concentration was maintained in the same manner as in the HMP (450 < pO_2_ < 550 mmHg). PVP, perfusion flow, bile production during perfusion, pO_2_, pCO_2_, and pH were directly measured and monitored by calculating the PVR and oxygen consumption rate (OCR).

### 2.7. Sample Collection

At the end of reperfusion, the livers were weighed and collected. Samples for adenine nucleotide and purine catabolite assays were collected by freeze-clamping and stored in the vapor phase of liquid nitrogen. For other assays, liver samples were stored at −80 °C until use. For histological evaluation, formalin-fixed paraffin-embedded sections were stained with hematoxylin and eosin (HE). Perfusates were collected at 5, 30, 60, 120, and 180 min (the end of perfusion) in the HMP and 5, 30, and the end of perfusion (90 min) in the IPRL. The activities of aspartate transaminase (AST), alanine transaminase (ALT), and lactate dehydrogenase (LDH) in the perfusate were measured using standard blood biochemistry assays based on the International Federation of Clinical Chemistry and Laboratory Medicine (IFCC) methods approved by Sapporo Clinical Laboratory Inc. (Sapporo, Japan).

### 2.8. IPRL Calculations

The PVR and OCR were calculated as previously described [22].

PVR [cmH_2_O × min × g liver/mL] = PVP (cmH_2_O)/portal flow (mL/min/g liver). Hepatic enzyme release (IU/g liver) = [Ct × V]/LW (g). Where Ct is the enzyme activity (mIU/mL) in the perfusion medium after t min of reperfusion, V (mL) is the perfusate volume (300 mL), and LW (g) is the liver weight before CS or HMP.

OCR (µmol O_2_/min/g liver) = (C_in_ − C_out_) × portal flow (mL/min/g liver), where C_in_ and C_out_ are the O_2_ concentrations in the inflow and outflow, respectively. O_2_ concentration (µmol O_2_/mL) = pO_2_ (kPa)·SO_2_ (37 °C) (µmol O_2_/mL/kPa), where SO_2_ (37 °C) is the oxygen solubility in water at 37 °C. SO_2_ (37 °C) = 0.01056 (µmol O_2_/mL/kPa).

### 2.9. Histology and Apoptotic Index

The formalin-fixed paraffin-embedded sections were subjected to HE staining and TdT-mediated dUTP nick end labeling (TUNEL) staining using the In Situ Apoptosis Detection Kit (Takara Bio. Shiga, Japan), according to the manufacturer’s instructions. Nuclear counterstaining was performed using hematoxylin. The apoptotic index was calculated as the ratio of TUNEL-positive hepatocytes per cell number in high-power fields (HPFs). The average of three HPFs represented each sample.

### 2.10. Scanning Electronic Microscopy (SEM)

Osmium maceration was performed as previously described [23]. Briefly, small pieces of the liver samples were pre-fixed in 0.5% paraformaldehyde and 0.5% glutaraldehyde in a phosphate buffer for 30 min on ice. After washing, the pre-fixed specimens were enclosed in amber glass bottles and sent to the co-investigator. The pre-fixed specimens were immersed in 1% osmium tetroxide (OsO_4_), washed in a phosphate buffer, immersed in 25 and 50% dimethyl sulfoxide, and frozen on a flat aluminum block. Then the specimens were immersed again in 0.1% OsO_4_ for 96 h at 20 °C to remove cytoplasmic proteins. The specimens were post-fixed in 1% OsO_4_, stained with tannic acid and 1% OsO_4_, dehydrated, lyophilized in an VFD-21S freeze-dryer (Vacuum Device Inc., Mito, Japan), mounted onto a metal plate, and coated with osmium using an HPC-1SW plasma device (Vacuum Device Inc., Mito, Japan) for evaluation using a Regulus 8100 scanning electron microscope (Hitachi).

### 2.11. Western Blot Analysis

The frozen tissues were minced and homogenized with a glass-Teflon homogenizer and subjected to nuclear, mitochondrial, and microsomal (cytoplasmic) fractionation using a LysoPure™ Nuclear and Cytoplasmic Extractor Kit with an inhibitor cocktail of proteases (1%) and phosphatases (1%). Briefly, the homogenate was centrifuged for 10 min at 600× *g* and 4 °C. The first pellet contained the nuclear fraction. The supernatant was centrifuged for 10 min at 15,000× *g* and 4 °C. The pellet contained a mitochondrial fraction. The resulting supernatant contained the microsomal (cytoplasmic) fraction. The protein concentration of each fraction was determined using a bicinchoninic acid assay (Thermo Scientific, Rockford, IL, USA).

Every 12 micrograms of the cytosolic or mitochondrial proteins were subjected to standard SDS polyacrylamide gel electrophoresis (SDS-PAGE) with 7.5% Mini-PROTEAN^®^ TGX Stain-Free™ Protein Gels (Bio-Rad Laboratories, Inc., Hercules, CA, USA), transferred onto a low fluorescent polyvinylidene difluoride (PVDF) membrane using Trans-Blot^®^ Turbo™ (Bio-Rad), and incubated with diluted primary antibodies (1:1000), pan-AMPK-alpha, phospho-AMPK-alpha, pan-SQSTM-1, phospho-SQSTM-1, ATG5, PARKIN, PINK-1, pan-JNK, and phospho-JNK overnight at 4 °C. The membranes were then incubated with horseradish peroxidase (HRP)-conjugated anti-rabbit (1:5000) or anti-mouse (1:1000) IgG secondary antibodies. Protein bands were detected using a ChemiDoc XRS^®^ chemiluminescent detector (Bio-Rad) and normalized using the total protein normalization method.

### 2.12. High-Performance Liquid Chromatography

Flavin mononucleotide (FMN), flavin adenine dinucleotide (FAD), and riboflavin (RF) in the perfusate of HMP were measured using a high-performance liquid chromatography (HPLC) method. The sample (perfusate) was deproteinized by mixing it with pure methanol, followed by centrifugation and filtration. An aliquot of the resulting deproteinized supernatant was directly injected into the HPLC system. The HPLC system consisted of a GASTORR BG-42 degasser (FLOM Co., Tokyo, Japan), L-7100 pump (Hitachi High-Tech Corporation, Tokyo, Japan), model 234 autoinjector (GILSON Inc., Middleton, WI, USA), ATC-10 column oven (Eicom, Kyoto, Japan), L-7400 UV detector (Hitachi), NOD-10 UV detector (Eicom), and F-1050 fluorescence spectrophotometer (Hitachi) with C18 column for reverse phase HPLC analysis InertSustain AQ-C18 (5 µm) and a guard column E (GL Science, Tokyo, Japan). The HPLC conditions were as follows: ODS column (EICOMPAK SC5-ODS; 3 µm, 150 × 4.6 mm), column oven (40 °C), UV–Vis detector (254 nm), fluorescence detector (excitation 445 nm, emission 530 nm), mobile phase (A. methanol, B. acetic acid buffer. A/B = 35/65, *v*/*v*), where the acetic acid buffer was a mixture of 4 M sodium acetate (20 mL) and 50% acetic acid (10 mL) in 1 L of deionized water; flow (0.7 mL/min), and injection volume (25 µL). FMN, FAD, and RF contents were expressed as mmol/L.

### 2.13. Statistical Analysis

Data were examined, excluding the control group, and were expressed as the mean ± SD (*n* = 6). We used one-way ANOVA and Dunnett’s tests to evaluate statistical significance compared with the CS or MP groups using JMP^®^ 15 (SAS Institute Inc., Cary, NC, USA). Statistical significance was set at *p* < 0.05.

## 3. Results

### 3.1. pH in the Perfusate during HMP

**Figure 1:**
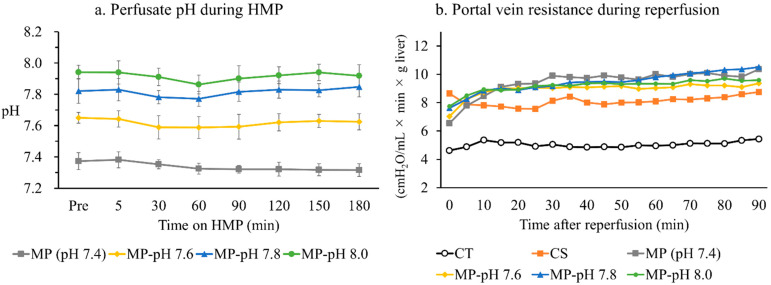
(**a**) Perfusate pH during HMP at 7–10 °C: Although the pH of UW-MPS^®^ was adjusted to 7.6, 7.8, and 8.0 before starting the HMP, it gradually dropped with perfusion. Therefore, the pH was adjusted by adding NaOH during the HMP. In contrast, the pH decreased in the MP Group; (**b**) Portal vein resistance during reperfusion at 37 °C The graft was perfused at a constant pressure (8 cmH_2_O in the CT group and 12 cmH_2_O in the other groups) on an IPRL. PVR maintained the lowest value in the CT group compared to the other groups throughout reperfusion. The PVR was 8.0 to 9.0 (cmH_2_O/mL × min × g liver) in the CS group and 9.0 to 10.0 (cmH_2_O/mL × min × g liver) in MP and MP-pH groups with no significant differences.

The pH of the perfusate in the HMP of the MP group without pH adjustment fluctuated, as shown in Figure 1a, at 7–10 °C. In the MP-pH groups with pH adjustment, the pH was adjusted, as shown in Figure 1a. 

### 3.2. Portal Venous Resistance during IPRL

In the control group, the PVR at each time point was maintained at a low value throughout the reperfusion, ranging from 4.0 to 5.0 (cmH_2_O/mL × min × g liver). The CS group showed a value of 8.0 to 9.0 (cmH_2_O/mL × min × g liver), whereas the MP and MP-pH groups showed values of 9.0 to 10.0 (cmH_2_O/mL × min × g liver) with no significant differences. (Figure 1b).

### 3.3. Liver Enzyme Leakage

The AST activity of the perfusate at 90 min after reperfusion was 10.5 ± 2.3 (×10^−1^ IU/g liver) in the CT group, increased to 35.6 ± 14.7 (×10^−1^ IU/g liver) in the CS group, and significantly reduced to 21.4 ± 8.1 (×10^−1^ IU/g liver) in the MP group. AST activities were significantly reduced in all the MP-pH groups compared to those in the CS and MP groups: MP-pH 7.6, 14.4 ± 2.3, MP-pH 7.8, 11.6 ± 2.9, MP-pH 8.0, and 17.5 ± 4.2 (×10^−1^ IU/g liver). Only the MP-pH 7.8 group appeared to show a significantly lower AST activity than the other MP groups.

The ALT activity of the perfusate at 90 min after reperfusion was 1.8 ± 1.2 (×10^−1^ IU/g liver) in the CT group, increased to 14.0 ± 6.9 (×10^−1^ IU/g liver) in the CS group, and significantly reduced to 6.2 ± 3.7 (×10^−1^ IU/g liver) in MP group. ALT activities were significantly reduced in all the MP-pH groups compared to those in the CS and MP groups as follows: MP-pH 7.6, 3.7 ± 1.8, MP-pH 7.8, 3.0 ± 0.7, MP-pH 8.0, and 5.4 ± 2.5 (×10^−1^ IU/g liver).

The LDH activity of the perfusate at 90 min after reperfusion was 1.57 ± 0.06 (×10^−1^ IU/g liver) in the CT group, increased to 58.10 ± 26.67 (×10^−1^ IU/g liver) in the CS group, and significantly reduced to 22.77 ± 14.08 (×10^−1^ IU/g liver) in the MP group. LDH activity was significantly reduced in all the MP-pH groups compared to those in the CS and MP groups (MP-pH 7.6, 14.7 ± 8.5; MP-pH 7.8, 17.1 ± 6.8; MP-pH 8.0, 25.1 ± 13.1 (×10^−1^ IU/g liver)).

### 3.4. Oxygen Consumption Rate

**Figure 2:**
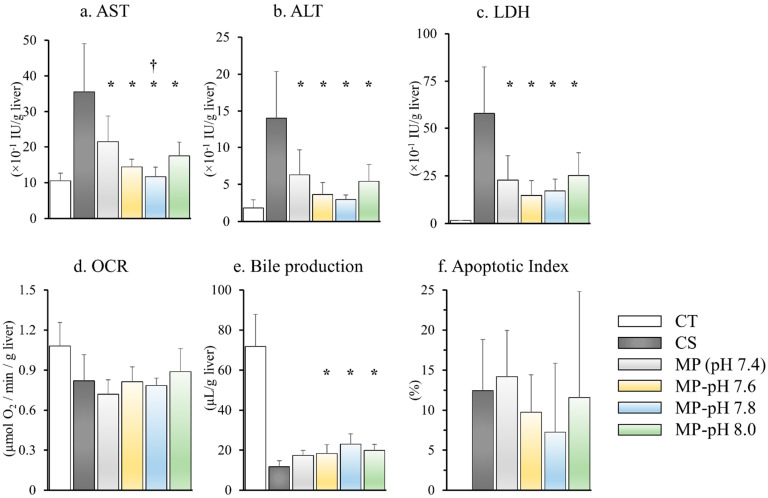
(**a**–**c**) Liver enzyme leakage in perfusate at 90 min after reperfusion. AST, ALT, and LDH in the MP and MP-pH groups were significantly lower than those in the CS group. Only AST in the MP-pH 7.8 group appeared to be significantly lower than that of the MP group. (**d**) OCR at 90 min after reperfusion. OCR was highest in the CT group. There were no significant differences between the CS and MP-pH groups. (**e**) Bile production during reperfusion: Bile production was highest in the CT group and significantly reduced in the CS group. Bile production in the MP-pH 7.6, 7.8, and 8.0 groups was significantly higher than that in the CS group. (**f**) Apoptotic index. TUNEL-positive cells were undetectable in the CT group. The TUNEL-positive cell ratio was increased in the CS and MP groups, whereas it was decreased in the MP-pH 7.8 group. Data are expressed as means ± SD (*n* = 6). *,†: A *p*-value less than 0.05 was considered significant: *: CS versus MP and MP-pH groups, †: MP versus MP-pH groups.

Mitochondrial respiratory function was determined using OCR (Figure 2d). The OCR at 90 min after reperfusion was the highest; 1.08 ± 0.19 (µmol O_2_/min/g liver) in the CT group, 0.82 ± 0.21 in CS group, 0.72 ± 0.12 in the MP group, 0.81 ± 0.12 in the MP-pH 7.6 group, 0.78 ± 0.06 in the MP-pH 7.8 group, and 0.88 ± 0.19 (µmol O_2_/min/g liver) in the MP-pH 8.0 group, with no significant differences.

### 3.5. Bile Production

Integrated liver function during IPRL was evaluated based on bile production. Bile production was the highest at 72.0 ± 17.6 (µL/g) in the CT group. It was significantly reduced to 11.76 ± 3.21 (µL/g) in the CS group, whereas it was significantly higher in all the MP-pH groups as compared to that of the CS group as follows: 18.3 ± 4.9 in the MP-pH 7.6 group, 23.0 ± 5.7 in MP-pH 7.8 group, and 19.8 ± 3.3 (µL/g) in MP-pH 8.0 group. Bile production in the MP group (17.3 ± 2.8 µL/g) was not statistically different from that of the CS group. In addition, bile production did not differ in the CS and MP-pH groups (Figure 2e).

### 3.6. Liver Histopathology

**Figure 3:**
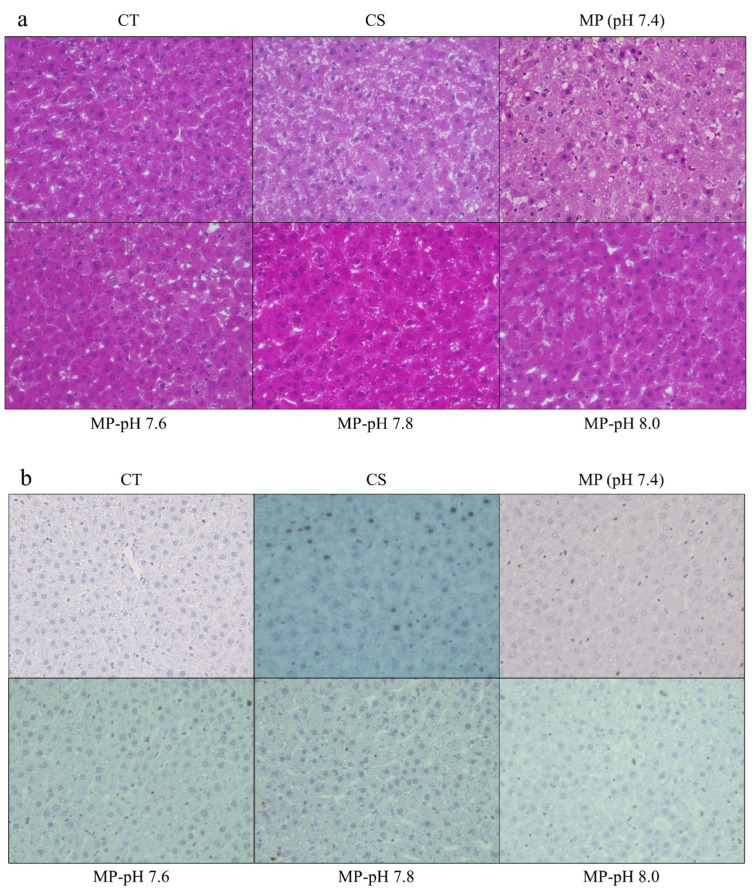
Liver histopathological examination at 90 min after reperfusion: (**a**) HE staining showed an almost normal appearance in the CT group. Vacuolization and condensed or swollen nuclei with heterogeneous staining were observed in the CS and MP groups, whereas these findings were suppressed in the MP-pH groups, especially at pH 7.8. (**b**) TUNEL staining showed the highest positive cell ratio in the CS group and the least in the CT group. The number of positive cells tended to decrease in MP-pH groups.

Liver histological examination 90 min after reperfusion showed an almost normal appearance in the CT group. Severe vacuolization and condensed or swollen nuclei with heterogeneous staining were observed in the CS and MP groups. In contrast, these findings were suppressed in the MP-pH groups, especially at pH 7.8 (Figure 3a).

### 3.7. Apoptosis

TUNEL-positive cells were not observed in the CT group. The TUNEL-positive cell ratio increased in the CS and MP groups, whereas it decreased in the MP-pH 7.8 group with no significant differences (Figure 2f and Figure 3b).

### 3.8. Scanning Electron Microscopy

**Figure 4:**
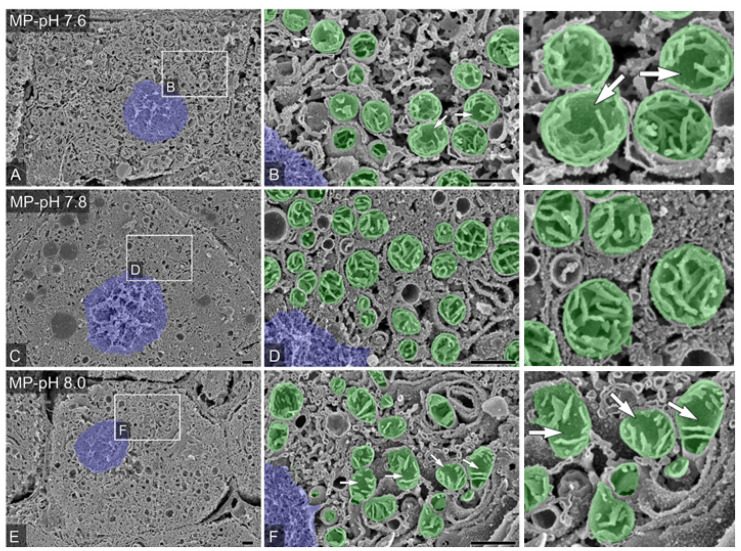
Liver scanning electron microscopy (SEM) findings at 90 min after reperfusion: The blue areas are nuclei, and the green areas are mitochondria. The mitochondria in the MP-pH 7.6 and pH 8.0 groups had pendulous, flat structures, few rod-like structures, and low cristae density (arrow). In contrast, rod-like structures and dense cristae were preserved in the pH 7.8 group. Single hepatocyte in MP-pH 7.6, 7.8, and 8.0 groups were presented, respectively (**A**,**C**,**E**), and subfigures (higher magnification; **B**,**D**,**F**). The photos in the right column are the enlarged version of the middle column. The scale bar in the figure represents 1 µm.

In the osmium-maceration SEM 90 min after reperfusion, the cell morphology of the CT group was well preserved, while in the CS group, there were several cells with numerous vacuolization, enormous mitochondria, and coarse cristae structures. The MP and MP-pH groups preserved the general structure of the cell under low magnification. However, there were differences in the density and architecture of the cristae between the groups when focused on the mitochondria. The mitochondria in the MP group had pendulous, flat structures, fewer rod-like structures, and low cristae density. Some of these cristae structures were observed in the MP-pH 7.6 and pH 8.0 groups. In contrast, rod-like structures and dense cristae were preserved in the pH 7.8 group (Figure 4).

### 3.9. Autophagy and Inflammatory Signals

**Figure 5:**
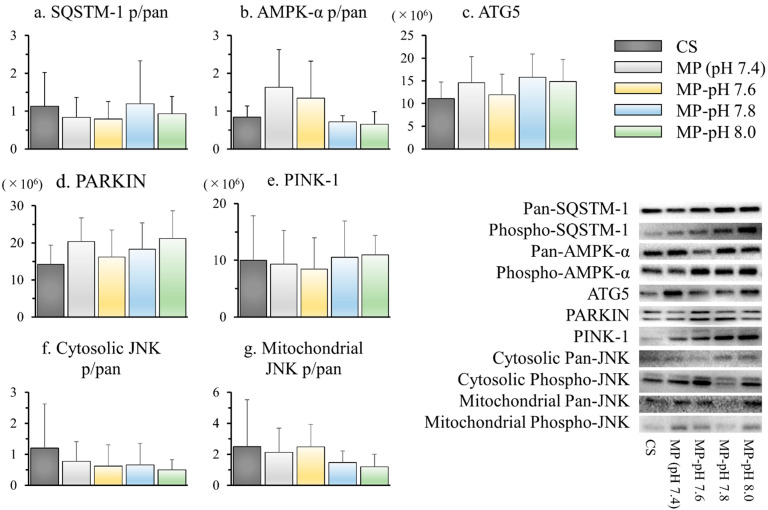
Autophagy and inflammatory signals by western blotting. The degree of phosphorylation was represented by the ratio of signal intensity derived from against phosphorylated and pan antibodies (p/pan). (**a**) AMPK-α (p/pan), (**b**) SQSTM-1 (p/pan), (**c**) ATG5, (**d**) PARKIN, (**e**) PINK-1, (**f**) cytosolic JNK (p/pan), and (**g**) mitochondrial JNK (p/pan). The band intensity was normalized by the total protein in (**c**–**e**). The representative photos were shown in the lower right. There were no differences among all groups for all signals.

Macro- and microautophagy were evaluated through measurements of cytosolic AMPK-α, SQSTM-1, ATG5, mitochondrial PARKIN, and PINK-1 using western blotting. AMPK-α and SQSTM-1 are denoted as phospho/pan, which indicates the ratio of the activated signal. ATG5, PARKIN, and PINK-1 represent normalized band intensities. There were no differences in any of the signals between the groups. Acute stress responses were evaluated by western blotting of the cytosolic and mitochondrial MKK4-JNK pathways. JNK is denoted by phospho/pan, as described above. There were no significant differences among the groups (Figure 5).

### 3.10. Vitamin B2

**Figure 6:**
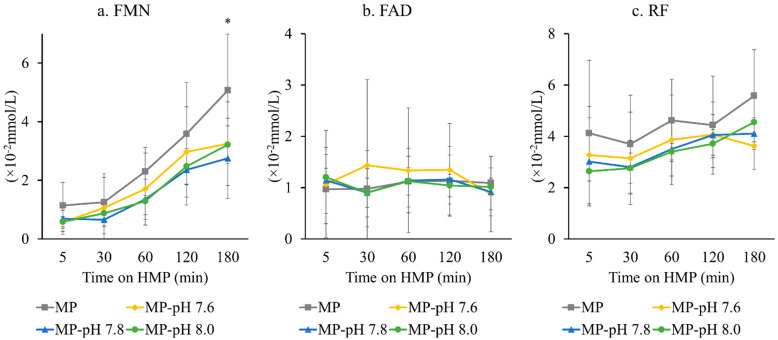
Vitamin B2 in the effluent. (**a**) FMN showed an increasing trend over time in all MP and MP-pH groups. MP-pH 7.8 showed a significantly lower value at the end of 180 min of HMP as compared to that of the MP group. (**b**,**c**) FAD and RF did not show any significant changes among the groups. Data are expressed as means ± SD (*n* = 6). *: A *p*-value less than 0.05 was considered significant: *: MP versus MP-pH groups.

FMN was measured in the effluent using HPLC as a marker of mitochondrial damage. FMN showed an increasing trend over time, with the highest value in the MP group and lower and similar values in the MP-pH 7.6, 7.8, and 8.0 groups. Perfusate FMN in the MP group was significantly higher than that in the MP-pH 7.8 group at the end of the 180 min of HMP (Figure 6a). Other vitamin B2 species detected using the same fluorescence wavelengths, FAD and riboflavin (RF), were measured. FAD and RF levels did not show statistically significant changes among the groups.

## 4. Discussion

In the current study, we investigated the optimal pH for liver HMP by adjusting the pH of the UW-gluconate solution. HMP for DCD liver is more effective than CS in reducing transaminase levels [24]. In addition, HMP at higher pH showed better protection, as evidenced by higher bile production, lower levels of transaminase concentrations during reperfusion, and the histopathological findings after reperfusion. Among the pH values evaluated, HMP at pH 7.8 appeared to be the most optimal option. These observations suggest that maintaining the acid-base balance within a permissive range during HMP resulted in the amelioration of post-reperfusion injury in the DCD liver.

The use of DCD livers as a solution to donor shortages in liver transplantation is attracting attention, and research has been conducted to suppress the progression of reperfusion injury associated with warm ischemia. A warm ischemic period, even for a short duration, profoundly affects the liver endothelial phenotype, making it dysfunctional, and leads to an immediate increase in hepatic vascular tone, inflammation, polymorphonuclear cell infiltration, and cell death in hepatocytes [25]. These reactions are complex and confounding and lead to intra- and extracellular acidosis. Acidosis further causes a mitochondrial imbalance in charge transfer and ROS production, leading to severe lesions during reperfusion, and compromises graft survival [26]. HMP has been validated as a countermeasure to ischemia-reperfusion injury [24,27,28]. It prevents the impairment of the mitochondrial electron transport chain and protects against subsequent reperfusion injury by inducing mitochondrial reprogramming by uploading the nucleotide pool and effective succinate metabolism [29,30,31]. However, the repair effect of HMP at pH 7.4 and 7–10 °C was insufficient in the present study, presumably due to the relatively acidic conditions. If we assume that the optimal pH for intracellular and extracellular environments at 37 °C is 7.200 and 7.400, respectively, and the pH changes according to the Rosenthal factor (−0.0147) [13], then it can be inferred that the pH values for intracellular and extracellular fluids at 7 °C would be 7.641 and 7.841, respectively. This finding is consistent with the results of the present study. With a thorough understanding of the pH changes in hypothermia and appropriate control of the pH, HMP can be used to further restrain reperfusion injury and fully exert its reparative effects.

The increase in FMN in the perfusate of HMP was suppressed in the pH-regulated HMP compared to that in the pH-unregulated one. In particular, significant suppression was observed at pH 7.8, which was the most effective HMP in this experiment, suggesting that the protective effect of HMP on mitochondrial electron transport chain complex I was enhanced by avoiding acidosis at 7 °C. Previous studies have reported that FMN release in HMP is associated with ischemia-reperfusion injury and is a marker of mitochondrial injury. However, in these studies, the fluorescence intensity was measured without separating the vitamin B_2_ species, FAD, FMN, and RF [29,30,31]. In this study, FAD and RF showed no differences between pH-regulated and unregulated HMP, but FMN showed a significant difference. These results suggest the legitimacy of FMN as a marker of mitochondrial injury during HMP. However, anticipated misjudgment due to the fluorescence derived from FAD and RF should be considered.

In the osmium-maceration SEM findings, differences in mitochondrial size and cristae, the mitochondrial inner membrane structure, were observed among the groups. The morphology of the mitochondrial inner membrane is important for the formation of proton gradients across the inner membrane for ATP synthesis, and increasing the density of the inner mitochondrial membrane alters the ATP production capacity of mitochondria [32,33]. External factors such as temperature also alter the mitochondrial size and area ratio of the inner and outer mitochondrial membranes [34], while internal factors such as lipid composition and unsaturation level alter energy production capacity [35]. Changes in various internal and external factors have been reported to dynamically regulate the structure and function of mitochondria, including the cristae, which is the site of mitochondrial oxidative phosphorylation. The mitochondrial membrane composition of fatty acids with high unsaturation that transport oxygen at high speed, fat droplet composition, and cytochrome oxidase expression levels are dynamically regulated. The present findings of mitochondrial observation by osmium-maceration SEM were consistent with these reports [32,33,34,35]. In addition, the establishment of a grading method to evaluate the length of cristae, mitochondria size, mitochondria-endoplasmic reticulum contact area, and mitochondria-lipid droplet contact area in a model of cardiac arrest organ damage caused by cold storage and cold perfusion can elucidate the mechanism of mitochondrial dysfunction described in this model and identify new therapeutic targets.

We explored mitochondrial repair or mitophagy based on scanning electron microscopy findings as a mechanism to suppress reperfusion injury. In damaged or dysfunctional mitochondria, PINK-1 accumulates in the outer mitochondrial membrane. PARKIN in the cytosol then migrates into the mitochondrial outer membrane and is activated, inducing mitophagy [36]. In this study, the absence of significant differences between all groups in mitochondrial PARKIN or PINK-1 in WB ruled out an enhancement of mitophagy by HMP, even at optimal pH. AMPK-α, SQSTM-1, and ATG5 were measured as proteins involved in macro-and microautophagy, in addition to mitophagy [37,38,39]. However, no significant differences were observed among all groups, negating the association between HMP-induced repair and autophagy. Similarly, no association was observed with the inhibition of injury in the JNK pathway [40,41].

A limitation of this study was the method used to control the pH of the UW-gluconate solution, into which NaOH, a strong base, was intermittently added. The pH adjustment method is complex. Thus, to minimize the fluctuation of perfusate pH during HMP, further investigation of buffer components, pH monitoring, and adjustment methods are necessary for the future development of MP. Another limitation is that the method of evaluation was an IPRL, which simulates reperfusion without blood components only for 90 min. It is necessary to evaluate this hypothesis in liver transplantation in both small and large animals.

## 5. Conclusions

In conclusion, we found that the most effective pH was 7.8 for HMP at 7 °C in DCD rat liver. At this pH, mitochondrial ultrastructure and function of the electron transport chain complex were well preserved, leading to reduced hepatic ischemia and reperfusion injury.

## Data Availability

The data presented in this study are available on request from the corresponding author. The data are not publicly available due to some ongoing analyses.
